# Differences in fatty acid profiles and desaturation indices of abdominal subcutaneous adipose tissue between pregnant women with and without PCOS

**DOI:** 10.1080/21623945.2019.1710021

**Published:** 2020-01-07

**Authors:** Neda Emami, AliReza Alizadeh, Ashraf Moini, Parichehreh Yaghmaei, Maryam Shahhosseini

**Affiliations:** a Department of Biology, Faculty of Science, Science and Research Branch, Islamic Azad University, Tehran, Iran; b Department of Embryology, Reproductive Biomedicine Research Center, Royan Institute for Reproductive Biomedicine, ACECR, Tehran, Iran; c Department of Endocrinology and Female Infertility, Reproductive Biomedicine Research Center, Royan Institute for Reproductive Biomedicine, ACECR, Tehran, Iran; d Breast Disease Research Center (BDRC), Tehran University of Medical Sciences, Tehran, Iran; e Department of Gynecology and Obstetrics, Arash Women’s Hospital, Tehran University of Medical Sciences, Tehran, Iran; f Reproductive Epidemiology Research Center, Royan Institute for Reproductive Biomedicine, ACECR, Tehran, Iran; g Department of Genetics, Reproductive Biomedicine Research Center, Royan Institute for Reproductive Biomedicine, ACECR, Tehran, Iran; h Department of Cell and Molecular Biology, School of Biology, College of Science, University of Tehran, Tehran, Iran

**Keywords:** Abdominal Subcutaneous Adipose Tissue, Fatty Acid Profiles, Polycystic Ovary Syndrome

## Abstract

The objective was to determine the differences in fatty acid (FA) profiles in subcutaneous adipose tissue (AT) between pregnant women with polycystic ovary syndrome (PCOS) and those without PCOS. FA profiles of AT samples from 13 PCOS and 32 non-PCOS, all of whom underwent caesarean section were compared using gas chromatography. Age and BMI in the two groups were similar. Twenty-one FAs were detected and the total saturated FA percentage of experimental groups was similar. While the total monounsaturated FA (MUFA) (p < 0.0004) and desaturase index (18:1 cis-9/18:0; p < 0.03) were higher in PCOS women than non-PCOS women, total polyunsaturated FA (PUFA) was lower in PCOS than non-PCOS women (p < 0.004). Docosahexaenoic acid level of the two groups was similar while α-linolenic acid and eicosapentaenoic acid levels were significantly (p < 0.05) lower in PCOS. Total trans-FA, C18:1 t9 and C18:2t were lower in PCOS women (p < 0.05). These results indicate differences in desaturase index, MUFA and PUFA, especially n-3 FA in AT between age and BMI-matched pregnant PCOS and non-PCOS pregnant subjects. Further studies are warranted to replicate these findings and to investigate potential changes in these profiles in non-pregnant PCOS women.

## Introduction

Polycystic ovary syndrome (PCOS) is the most common endocrine disorder in women and is estimated to affect 6–10% of women of reproductive age [1]. PCOS is considered the foremost of ovarian diseases and has therefore been studied in detail. Not only PCOS patients can present a wide range of signs and symptoms, which make difficult the precise grading of the condition, but it is also associated with a variety of factors, including menstrual irregularity and lipid metabolism disorders. Therefore, the aetiology of PCOS is not completely understood yet and PCOS is considered a multifactorial disorder with various genetic, metabolic, endocrine and environmental abnormalities.

Several genetic and environmental parameters have been recognized to be effective factors in PCOS. Recently, Foley and Marsh recommended studies that can help understand the relationship between lifestyle and PCOS with the aim of streamlining the approach to symptom management [2]. In addition to psychosocial stress factor roles, they suggested that dietary components that are highly processed and enriched with simple carbohydrates and saturated fats can be used as a starting point for highlighting foods that must be avoided in PCOS diets. Indeed, the importance of fatty acids (FA) was reported and it seems that adipose tissue (AT) was the best indicator for FA status. Since the half-life of fat is 2 y, analyses of AT can indicate FA levels over a relatively long period. On the other hand, the role of genes is irrefutable in PCOS and it is worth highlighting that few studies confirm the differences in gene expression related to FA metabolism in AT of PCOS women [3]. Although AT has crucial roles in PCOS, very little information exists on FA profiles in AT of PCOS women. Therefore, more investigations on effective several factors including AT roles in PCOS are necessary. [4]

It has been suggested that AT may involve in PCOS and androgen excess was closely related to the lipid metabolism disorder. Actually, AT is not only recognized as a fat storage organ but is also regarded as an organ with important endocrine and metabolic functions [5] and subcutaneous fat biopsies can be used as an integrated long-term indicators of FA metabolism [6–8]. Previously, the importance of FA profiles in AT of pregnant women and mothers has been shown by researchers from France [9], Poland [10], and Portugal [11], which warrants further studies in developing countries such as Iran. On the other hand, it has been suggested that FAs are associated with the risk of a wide range of chronic diseases such as PCOS [12,13]. In this regard, trans-FA (TFA) and saturated FA (SFA) are reported to play significant roles in the development of several disorders through their stimulating action and n-3 polyunsaturated fatty acids (PUFA) contribute by their suppressive action [11,13–15]. The increased amounts of linoleic acid (LA; C18:2 n-6) by 136% in the AT over the last half century in the United States [16], and the increase in the dietary n-6:n-3 FA ratio from 1:1 to 20:1 [17], point to their possible roles in chronic diseases such as PCOS. Furthermore, given multiple roles of monounsaturated fatty acids (MUFAs), variations in stearoyl-CoA desaturase (SCD1) activity in mammals would be expected to affect a variety of key physiological variables. SCD1 is an endoplasmic reticulum enzyme that catalyzes the biosynthesis of MUFA from SFA that are either synthesized *de novo* or derived from the diet [18]. The enzyme activity was illustrated by delta-9 desaturase index which was calculated using the MUFA: SFA ratio. Interestingly, Corton et al. [3] reported that *SCD*gene expression is elevated in abdominal subcutaneous AT of PCOS women but they did not report the FA profiles and desaturation index in subjects.

To the best of our knowledge, differences in AT’s FA profiles between PCOS and non-PCOS pregnant women have not been addressed yet. It is important to understand the relationship between PCOS and FA profiles in the AT of pregnant women, for the wellbeing and appropriate care of the mother and her baby. Towards gaining this understanding, the current study was designed to determine differences in FA profiles and desaturation indices in abdominal subcutaneous AT in 45 PCOS and non-PCOS Iranian pregnant women.

## Materials and methods

### Subjects and adipose tissue biopsies

After obtaining permission from the Royan Institute ethics committee, Tehran, Iran (IR.ACECR.ROYAN.REC.1398.087), samples and demographic data were collected from three hospitals in Tehran, Iran. This study involved 45 Iranian pregnant women who underwent caesarean section including 13 PCOS and 32 non-PCOS cases. Samples were taken from subcutaneous fat of abdomen. Signed informed consent was obtained from all subjects. During the sampling time, the following information was collected: age, weight (before pregnancy and at delivery day), height, parity, gestational duration, smoking, specific nutritional habits, alcohol drinking and using a written questionnaire. In keeping with the local guidelines, all mothers were provided standard diet during pregnancy as recommended by the Iranian Ministry of Health guidelines. According to 2003 Rotterdam criteria [4], the diagnostic traits of PCOS are the presence of two or more significant symptoms of the syndrome and patients that met two of three symptoms were chosen. PCOS diagnosis was done by the medical practitioners affiliated to Royan Institute. Intake of any medication affecting glucose and lipid metabolism, especially FA supplement and being diabetic, alcoholic, and smoker was defined as exclusion criteria. Reasons for delivery by caesarean section included maternal choice (<10%) and previous caesarean delivery, diagnosis of medical practitioner and others.

At the time of caesarean section, the surgeon took 3–4 g subcutaneous fat upon entering the abdomen. Immediately, biopsies were washed in isotonic saline solution, segmented, floated in liquid nitrogen, placed in cryovial tubes and snap-frozen as described previously [19,20]. Samples were stored in liquid nitrogen (−196°C) until analysis.

### Fatty acid analysis

Samples were thawed at room temperature for 2 min, segmented and immediately methylated using 0.5 ml of 0.2 N metabolic sodium hydroxide for 30 min. The process was then continued using 1 ml of 14% boron trifluoride in methanol (BF3-methanol) at room temperature for about 30 min. Reconstitution of FA methyl esters was carried out in 1 ml of hexane [21]. Gas chromatography (Shimadzu, Nexis 2030, Japan) with a 100 m (0.25 mm I.D.) capillary column (Dikmacap-2330) was utilized to determine the concentration of FA. Hydrogen was used as the carrier gas, and the prime and ultimate temperatures and detector and injector temperatures were set at 170, 230, 300 and 260°C, respectively.

### Statistical analysis

Data were initially tested for normal distribution using Kolmogorov–Smirnov test. Data with normal distribution were analysed using the *t* test. Data that did not have normal distribution were analysed using Mann–Whitney U test. All analyses were conducted in SAS. Differences were considered significant at P ≤ 0.05.

## Results

**Figure 1.**
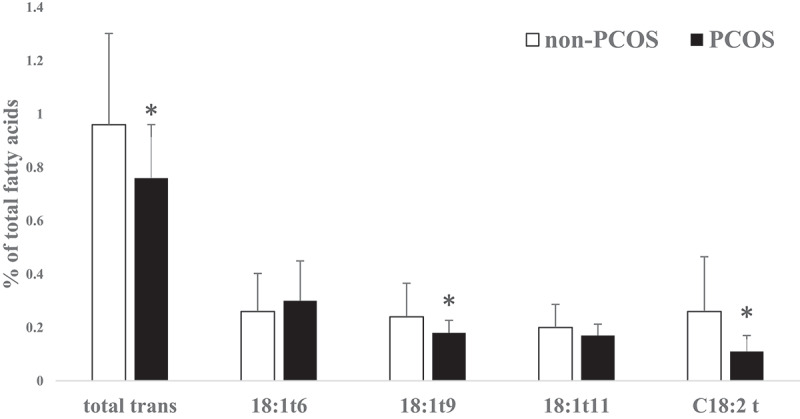
Trans-fatty acid differences in subcutaneous adipose tissue of pregnant non-PCOS (n = 32) versus PCOS women (n = 13) (Mean ± sd)(* total trans: *p = 0.021*; C18:1 t9: *p = 0.044* and C18:2:*P = 0.0005*)

**Figure 2.**
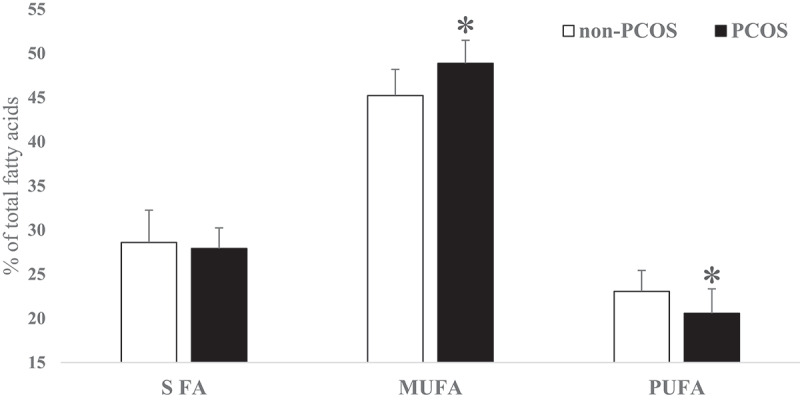
Saturated fatty acid (SFA), Monounsaturated fatty acid (MUFA) and Polyunsaturated fatty acid (PUFA) differences in subcutaneous adipose tissue of pregnant non-PCOS (n = 32) versus PCOS women (n = 13) (Mean ± sd) (* p < 0.01)

No significant differences were found in age and body mass index (BMI) between non-PCOS and PCOS women. The FA profiles of subcutaneous AT in PCOS and non-PCOS groups are presented in Table 1. Saturated FA, unsaturated FA, odd chain FA, n6:n3 ratio and saturated:unsaturated ratio were not significantly different between non-PCOS and PCOS women. Palmitic acid (16:0) did not vary significantly between the two groups (p > 0.05) and it was found to be the most prevalent SFA in subcutaneous AT. Oleic acid (C18:1 n-9 cis) was the most prevalent monounsaturated FA (MUFA) which was significantly (P = 0.0007) higher in PCOS women. Moreover, the desaturase index for C18:1cis/C18:0 was significantly (P = 0.035) higher in PCOS women while the differences in C16:1cis/C16:0 ratio were not significant (Table 2). As the main component of PUFA, linoleic acid (18:2 n-6 cis) was significantly (P = 0.006) lower in PCOS women. Docosahexaenoic acid (DHA) concentration was not significantly different between the two groups while total n-3 FA (P = 0.001), α-linolenic acid (ALA) (P = 0.016) and eicosapentaenoic acid (EPA) (P = 0.003) were significantly lower in PCOS women. Total trans-FA (P = 0.021), C18:1 t9 (P = 0.044) and C18:2 t (P = 0.0005) were lower in PCOS women. However, C18:1 t11 as well as C18:1 t6 concentrations were not significantly different between PCOS and non-PCOS women (Table 1 and Figure 1). In all, total SFA was not significantly different between the two groups. Total MUFA content was significantly (P = 0.0004) higher in PCOS than non-PCOS women (49 vs. 45%, respectively). In contrast, total PUFA was significantly (P = 0.004) lower in PCOS than non-PCOS women (20 vs. 23%, respectively; Figure 2).

**Table 1.** General characteristics of study participants (PCOS: polycystic ovary syndrome and non-PCOS) and fatty acid profiles in experimental groups (Means ± sd)

	non-PCOS (n = 32)	PCOS (n = 13)	P-Value
Age (year)	31.5 ± 5.42	31.4 ± 3.57	NS
BMI (kgm^−2^) before pregnancy	26.5 ± 4.93	26 ± 5.92	NS
BMI (kgm^−2^) at delivery day	31 ± 5.33	30.1 ± 5.35	NS
Fatty Acids (% of total fatty acids)			
C 12:0	0.29 ± 0.16	0.26 ± 0.12	NS
C 14:0	1.67 ± 0.46	1.92 ± 0.47	NS
C 15:0	0.23 ± 0.1	0.30 ± 0.16	NS
C 16:0	22.2 ± 2.59	21.73 ± 1.85	NS
**C 17:0**	**0.30 ± 0.15**	**0.41 ± 0.17**	**0.040**
C 18:0	3.89 ± 1.15	3.29 ± 0.89	NS
C 14:1	0.19 ± 0.11	0.22 ± 0.12	NS
C 15:1	0.09 ± 0.12	0.04 ± 0.05	NS
C 16:1	3.64 ± 0.78	4.34 ± 1.37	NS
C 18:1t6	0.26 ± 0.14	0.30 ± 0.15	NS
**C 18:1t9**	**0.24 ± 0.13**	**0.18 ± 0.05**	**0.044**
C 18:1t11	0.20 ± 0.09	0.17 ± 0.04	NS
**C 18:1c**	**40 ± 2.47**	**43 ± 2.07**	**0.0007**
**C 18:2c**	**21.3 ± 2.23**	**18.9 ± 2.77**	**0.005**
**C 18:2t**	**0.26 ± 0.2**	**0.11 ± 0.06**	**0.0005**
**C 18:3 n-3**	**0.78 ± 0.13**	**0.68 ± 0.12**	**0.016**
C 20:1	0.62 ± 0.23	0.56 ± 0.10	NS
C 20:2	0.29 ± 0.18	0.34 ± 0.06	NS
**C 20:4 n-6**	**0.30 ± 0.18**	**0.40 ± 0.09**	**0.021**
**C 20:5 n-3 (EPA)**	**0.06 ± 0.04**	**0.02 ± 0.03**	**0.003**
C 22:6n3 (DHA)	0.09 ± 0.04	0.08 ± 0.04	NS
Other fatty acids	3.12 ± 1.16	2.64 ± 0.87	NS
Total Saturated fatty acids	28.6 ± 3.66	27.9 ± 2.37	NS
Total Unsaturated fatty acids	68.3 ± 3.60	69.5 ± 2.31	NS
**Total MUFA**	**45.2 ± 2.96**	**48.88 ± 2.61**	**0.0004**
**Total PUFA**	**23 ± 2.37**	**20.57 ± 2.77**	**0.004**
**Total n-6 fatty acids**	**23.1 ± 2.29**	**19.80 ± 2.73**	**0.006**
**Total n-3 fatty acids**	**0.93 ± 0.15**	**0.77 ± 0.12**	**0.001**
n-6/n-3 ratio	24.1 ± 3.50	25.97 ± 4.31	NS
**Total trans fatty acids**	**0.96 ± 0.34**	**0.76 ± 0.2**	**0.021**
Odd chain FA	0.62 ± 0.24	0.74 ± 0.23	NS

NS; Non-significant; t: trans; c: cis; EPA: eicosapentaenoic acid; DHA: docosahexaenoic acid; BMI: Body mass index; MUFA: Monounsaturated fatty acid; PUFA: Polyunsaturated fatty acid. Fatty acids in bold were significant.

**Table 2.** Desaturase index (monounsaturated fatty acid/saturated fatty acid) in subcutaneous adipose tissue of pregnant PCOS (Polycystic ovary syndrome) and pregnant non-PCOS women (Mean ± sd). Desaturase index in bold was significant

	non-PCOS (n = 32)	PCOS (n = 13)	P-Value
C16:1 cis/C16:0	0.17 ± 0.05	0.20 ± 0.06	0.057
**C18:1 cis/C18:0**	**11.3 ± 3.73**	**14 ± 4.14**	**0.035**

## Discussion

Adipose tissue provides a good handle on the long-term metabolism of FA. It was reported that FA profiles of white AT in pregnant women reflect maternal lipid metabolism over the last 6–9 months [9,11]. It may also reflect the specific metabolic adjustment that occurs during pregnancy [22]. Although overweight and obesity were recognized by BMI as representative of the quantity of AT, it is surprising to note that recent studies highlight the importance of other parameters such as body composition measurements [23] and FA profiles [8] as representative of the quality of AT. In this regard, body fat percent and body muscle percent as well as AT FA profiles have been suggested for analysis of fat profile. This study provides compelling evidence of differences in FA profiles in abdominal subcutaneous AT between PCOS and non-PCOS pregnant women.

There are little data available on FA profiles in AT of pregnant women in Asia, and to the best of our knowledge, no study has been done in this field in Iran. We found that the mean percentage of SFA did not vary significantly between the two groups. In an earlier study, the SFA percentage was reported to be 27% in AT of healthy French new mothers [9] 5 d after delivery which it was similar to our findings in both groups (28.6%). However, Boué et al. [24] reported that SFA in French women’s abdominal subcutaneous AT was 33% whereas in Portuguese pregnant women [11], it was 23%. AT content appears to be a promising and invaluable index for FA such as SFA that are less likely to be mobilized during pregnancy. It was suggested that Iranians may have a higher intake of carbohydrates in their diet which is reflected by high levels of SFA in AT. Sharifi and Amani [25] reported that total daily energy intake derived from carbohydrates was almost 50% in western diet while in Iranian diet, approximately 60% or more of total energy intake was provided by carbohydrates. However, Garaulet et al. [26] declared that SFA concentration in abdominal subcutaneous AT of American men and women was 27% which was close to the findings of Martin et al. [9] on French new mothers and the present study in Iran. Finally, it seems that our SFA data were in line with previous studies and it was surprising to note that PCOS and non-PCOS women have similar SFA concentrations in abdominal subcutaneous AT.

The mean concentrations of total n-6 FA including mainly linoleic acid (LA; C18:2 n-6) were higher in non-PCOS women. Zhao et al. [27], who focused on plasma metabolomics analysis, demonstrated that LA concentrations in the plasma of PCOS women were higher than healthy ones. In a comprehensive study, Guyenet and Carlson [16] illustrated that the mean concentration of LA in abdominal subcutaneous AT in studies done in the US was 9% during the 1960s and 1970s. Parallel with LA elevation in abdominal subcutaneous AT during the 20th century, London et al. [7] recorded 17% LA in Postmenopausal abdominal AT. Similarly, LA concentration in abdominal subcutaneous AT was reported to be 15% [26]. Likewise, previous studies done in Europe during the last decade of 20th century among French women and Spanish men and women found LA concentrations of 14% and 16%, respectively, in subcutaneous abdominal AT.

Given the problems of high LA levels, several countries all over the world took steps to reduce the amount of LA in AT [15,16]. Although we could not find similar data in Iran during the last years, increasing LA concentration reaching 21% in abdominal subcutaneous AT has been identified. Our findings in both groups support the theory [28] that the developing world is particularly vulnerable to the widespread adoption of high LA oils because of their low cost and high-quality taste and food performance. Therefore, it may warrant more attention in Iran. The elevation in LA of AT in Iran is also reflected by higher n-6:n-3 ratio. A high proportion of n-3 and low n-6 FA in tissues may be beneficial for health [15]. This has led to the concept of n-6:n-3 ratio and a low ratio is often used to indicate a favourable balance of FA.

LA elevation is critical in pregnant and lactating women [29] as well as animals. Brenna [28]in a comprehensive review done on 65 animal studies concluded that high LA/low ALA (high n-6:low n-3) seed oils fed as the exclusive source of fat to pregnant and lactating primates, mice, rats, and other species leads to biochemical, neural, visual, and behavioural abnormalities in the offspring. In fact, there is a competition between LA and ALA for conversion to longer-chain fatty acid and higher LA:ALA ratio is associated with a reduction of n-3 long chain PUFA (LCPUFA) synthesis [30]. Thus, high levels of LA concentration and high n-6:n-3 ratio in abdominal AT of PCOS as well as non-PCOS groups show that more attention must be paid to reducing n-6 sources and increasing n-3 sources in developing countries such as Iran.

The content of EPA and ALA was significantly higher in non-PCOS subjects. During pregnancy and lactation, there is a higher metabolic requirement for n-3 LCPUFA compared to non-pregnant women. Partially the n-3 LCPUFA status of the mother (i.e. levels in the maternal circulation), which can be mobilized from AT reservoirs, is the major determinant of foetal DHA and EPA supply [29]. It was suggested that androgen excess was closely related to the lipid metabolism disorder [31] and Phelan et al. [32] reported that greater plasma n-6 concentration and n-6:n-3 ratio are associated with higher circulating androgens. In the current study, the non-PCOS group showed higher LA concentrations while increased arachidonic acid (C20:4 n-6) (as a product) in PCOS supports previous findings. Finally, low levels of n-3, especially EPA, higher levels of n-6 PUFA as well as performance impairment in desaturase activity in PCOS women [33] may influence ALA and EPA concentrations in PCOS subjects.

Interestingly, our study indicated alterations in MUFA metabolism in PCOS women, which can be related to a previous report [3] on changes in delta-9 desaturase enzyme activity. SCD1 is the predominant enzyme isoform in human’s lipogenic tissues and is a delta-9 desaturase enzyme, responsible for the conversion of SFAs to MUFAs. Desaturase Indices (MUFA:SFA ratio) were used to understand delta-9 desaturase enzyme activity. *SCD1* in AT preferentially converts palmitate (PA; 16:0) and stearate (SA; 18:0) to palmitoleate (PMA; 16:1 n-7) and oleate (OA; 18:1 n-9), respectively [34]. A higher value of desaturase index in PCOS subjects seems to be associated with a few metabolic and reproductive features of PCOS. Sessler et al. [35] reported that PUFA represses the expression of *SCD1*gene in adipocytes by reducing the stability of *SCD1*mRNA. Oestrogen generally antagonizes *SCD1* expression, as evidenced by the ability of oestrogen administration to reduce human AT’s *SCD1* expression and decrease SCD1 expression in high-fat-fed mice [34]. Higher desaturase index in PCOS women was observed together with high levels of oleic acid percentage in AT of PCOS compared to non-PCOS women. Uniquely, Corton et al. [3] reported that non-pregnant PCOS women had up-regulation of *SCD1* expression in abdominal subcutaneous AT compared to non-PCOS ones, which was consistent with our FA profile data. While they suggested that this finding is a result of the study design and younger PCOS women (31 y) than non-PCOS subjects (40 y), our FA profile data provide evidence that desaturase index may not be affected by age and it was higher in PCOS women. Since the age mean values were similar between the two groups of the current study, desaturase index elevation in PCOS confirmed that changes in *SCD1* function are independent of age, and this notion warrants further studies for PCOS women.

While some dietary trans-FAs are known to have adverse health effects, especially during pregnancy [36], little information exists on the concentration and effects of trans-FA in AT samples. The function of an individual, especially trans C18:1 isomers, differs considerably between industrial (C18:1 t9; produced via partial hydrogenation of vegetable oils, margarine and fast foods) and natural (C18:1 t11 and C18:1 t6; found in dairy product) sources. In this regard, de Souza et al. [37] in a meta-analysis study concluded that while dietary natural trans-FA has beneficial effects on health, industrial trans-FA is harmful. Indeed, trans-FAs have to be distinguished according to their origin (ruminant or industrial) in order to understand their possible influence on foetal development and human health [36]. Our study not only measured total trans-FA percentage in abdominal AT of Iranian mothers but also analysed individual isomers of trans C18:1 in PCOS and non-PCOS women, for the first time. Our data confirm lower industrial trans isomer in abdominal subcutaneous AT of PCOS mothers. Similarly, Boué et al. [24] reported that C18:1 t9 percentage was 0.27 in French women abdominal subcutaneous AT. There is an important issue for trans-FA in AT and it seems that the sampling region may affect trans-FA levels in AT.

Accumulation of trans-FA may be found in buttock subcutaneous AT. It was reported that Iranian men and women have the highest level of total trans FA (~8% of total fatty acid) in buttock subcutaneous AT [38] even higher than values reported from the US (~4%) [7]and CostaRican (~2%) [39] studies. The idea of the levels of different trans-FA isomers in different AT’s body regions being biomarkers is an exciting and emerging area for research and clinical studies.

Our study has a few limitations that must be considered when interpreting our results. The present results were obtained for pregnant PCOS women and we need more investigations in AT for non-pregnant women with PCOS. Moreover, FA composition of AT is an established long-term biomarker FA status, but AT samples are not easily available. Therefore, an additional limitation is the small sample size of this study and additional studies could confirm these results in pregnant and non-pregnant PCOS women as well as mothers in several developing countries.

## Conclusion

The current study showed that pregnant women suffering from PCOS have different MUFA and PUFA profiles in abdominal subcutaneous AT versus age-matched and BMI-matched non-PCOS women. The SFA levels are similar among experimental groups. The results of the multivariate approach, which included desaturation indices along with the entire major FA of abdominal subcutaneous AT, support the hypothesis of desaturase enzyme disturbance and MUFA accumulation in PCOS women. Moreover, we showed a decrease in n-3 in AT of PCOS mothers than non-PCOS mothers, which must be considered in PCOS maternal and child nutrition areas.
